# Neuropathologic features of cognitively defined Alzheimer's disease subgroups in the Adult Changes in Thought study

**DOI:** 10.1002/alz.71938

**Published:** 2026-10-25

**Authors:** Caitlin S. Latimer, Rod L. Walker, Marika Bogdani, C. Dirk Keene, Amber L. Nolan, Daniel D. Child, Shubhabrata Mukherjee, Seo‐Eun Choi, Michael L. Lee, Connie Nakano, Kelly Ehrlich, Linda K. McEvoy, Paul K. Crane

**Affiliations:** ^1^ Department of Laboratory Medicine and Pathology University of Washington Seattle Washington USA; ^2^ Kaiser Permanente Washington Health Research Institute Seattle Washington USA; ^3^ Department of Pathology University of California, San Diego San Diego California USA; ^4^ Department of Medicine University of Washington Seattle Washington USA

**Keywords:** Adult Changes in Thought study, Alzheimer's disease cognitive subgroups, neuropathology

## Abstract

**BACKGROUND:**

Individuals with Alzheimer's disease (AD) dementia can be subgrouped based on cognitive performance at diagnosis. We compared neuropathology across cognitively defined AD subgroups in the Adult Changes in Thought study.

**METHODS:**

Scores for memory, executive functioning, language, and visuospatial abilities were derived from the Cognitive Abilities Screening Instrument and a neurocognitive battery. Subgroups were defined by relative performance within domains. Standardized neuropathologic evaluation was performed *post mortem*. Associations were estimated using ordinal logistic regression, with odds ratios (OR) and 95% confidence intervals (CIs) comparing neuropathology between the AD subgroups and relative to individuals without dementia.

**RESULTS:**

Of 885 participants with *post mortem* neuropathology, 370 had AD dementia: 54% AD–no domain, 15% AD–memory, 15% AD–visuospatial, 8% AD–language, and 7% AD–executive. Among AD subgroups, associations were detected for limbic‐predominant age‐related transactive response DNA binding protein 43 kDa encephalopathy neuropathologic change (LATE‐NC; *P* = 0.019) and hippocampal sclerosis (HS; *P* = 0.006), with highest levels in AD–language (LATE‐NC OR 2.37, 95% CI 1.03–5.46 and HS OR 2.19, 95% CI 0.88–5.50) and AD–memory (LATE‐NC OR 1.88, 95% CI 1.02–3.46 and HS OR 3.11, 95% CI 1.56–6.20) relative to AD–no domain, and lowest levels in AD–visuospatial. AD–visuospatial tended to have lowest estimated Alzheimer's disease neuropathologic change (ADNC) burden across AD subgroups. Relative to participants without dementia, all AD subgroups expectedly had higher ADNC burden, and several AD subgroups had higher odds of Lewy body pathology (*P* = 0.017) and gross infarcts (*P* = 0.004), with broadly similar estimates across multiple AD subgroups.

**DISCUSSION:**

Neuropathologic patterns of ADNC and co‐pathologies across AD dementia subgroups suggest cognitive phenotyping may inform clinicopathologic studies of AD dementia.

## Highlights

- Cognitively defined Alzheimer's disease (AD) subgroups differ in select neuropathologies.
- Limbic‐predominant age‐related transactive response DNA binding protein 43 kDa encephalopathy (LATE) and hippocampal sclerosis vary across AD cognitive subgroups.
- AD–visuospatial shows lower estimated AD and LATE pathology burden.
- AD–memory and AD–language show higher LATE and hippocampal sclerosis burden.
- Cognitive subtyping may inform clinicopathologic heterogeneity in AD.

## 1 BACKGROUND

Alzheimer's disease (AD) is a common age‐related neurodegenerative disease with a characteristic clinical presentation and defining pathologic features. Recently, there has been an increased emphasis on the biologic substrate of AD, which is pathologically defined by the presence of extracellular amyloid beta (Aβ) plaques and intraneuronal tangles of hyperphosphorylated tau (neurofibrillary tangles [NFTs]) in a stereotypical pattern throughout the brain.^1^, ^2^ Although the advent of neuroimaging and biofluid biomarkers has aided in the *ante mortem* identification of this pathology in recent years,^3^, ^4^ clinical diagnosis of AD dementia is still critical to patient management and research efforts to better understand the clinical–pathologic correlations in AD. Throughout this article, we use “AD dementia” to refer to the clinical syndrome of AD dementia and “ADNC” (AD neuropathologic change) to refer to its defining neuropathologic hallmarks per National Institute on Aging–Alzheimer's Association (NIA‐AA) criteria; “AD” alone is used only when referring to the disease entity in general terms.

AD dementia is defined as an amnestic syndrome.^5^ Nevertheless, there is considerable heterogeneity in patterns of cognitive impairment across domains at the time of AD dementia diagnosis.^6^, ^7^ Cognitive test data used to inform a clinical diagnosis of AD dementia can also be used to further characterize relative performance across specific cognitive domains. We have previously described our approach that uses memory, executive functioning, language, and visuospatial measures to subtype individuals with AD dementia based on impairments in specific cognitive domains relative to others.^8^, ^9^, ^10^, ^11^, ^12^ This approach defined four subgroups marked by a single domain that is relatively more impaired than others (AD–memory, AD–visuospatial, AD–language, and AD–executive function), one subgroup of multiple domains with relative deficits (AD–multiple domains), and one subgroup with no domain with relative deficits (AD–no domain, i.e., all domains similarly impaired). These subgroups are associated with distinct genetic markers,^12^ imaging characteristics,^9^ and different comorbidities.^13^

It is important to correlate *ante mortem* clinical data with the *post mortem* neuropathology because AD is pathologically heterogeneous, and individuals with a clinical diagnosis of AD dementia are found to have a wide range of ADNC upon neuropathologic examination. This includes the extent of amyloid plaque pathology (Thal phase), the extent of NFT pathology (Braak stage), and the density of neuritic amyloid plaques (Consortium to Establish a Registry for Alzheimer's Disease [CERAD] score).^1^, ^2^, ^14^, ^15^ While it has been shown that the greater the ADNC burden, the more likely an individual is to have had a dementia syndrome during life (and most typically AD dementia),^15^ it remains unknown whether cognitive AD subgroups show differential associations with measures of amyloid and tau pathology.

Neuropathologic examination has also revealed that clinical AD dementia is often associated with additional co‐pathologies.^16^, ^17^, ^18^, ^19^, ^20^, ^21^, ^22^ In fact, it is much more common to find multiple neuropathologies upon *post mortem* brain examination than to find the Aβ plaques and NFTs alone. Frequently identified co‐pathologies include Lewy bodies, aggregates of hyperphosphorylated transactive response DNA binding protein 43 kDa (pTDP‐43) in limbic‐predominant age‐related TDP‐43 encephalopathy (LATE), and vascular pathologies (microinfarcts, gross infarcts, cerebral amyloid angiopathy, and arteriolosclerosis). Although there are no biomarkers to definitively diagnose these pathologies during life, they do have characteristic clinical signs and symptoms that can hint at their presence.^23^, ^24^, ^25^, ^26^, ^27^, ^28^ However, it is unknown if this pathologic heterogeneity correlates with the clinical heterogeneity of cognitively defined AD dementia subgroups.

Here, we leverage extensive cognitive and neuropathological data from 885 participants in the Seattle‐based Adult Changes in Thought (ACT) study brain‐donation cohort to characterize the neuropathological features of cognitively defined subgroups in participants with a clinical diagnosis of AD dementia.

**RESEARCH IN CONTEXT**

1. **Systematic review**: A review of the literature (e.g., PubMed) identified prior work describing clinical, imaging, and genetic heterogeneity across cognitively defined Alzheimer's disease (AD) subgroups. While these subgroups have been associated with distinct atrophy patterns and clinical features, there are limited data examining whether they differ in *post mortem* neuropathology, particularly with respect to co‐pathologies such as limbic‐predominant age‐related transactive response DNA binding protein 43 kDa encephalopathy (LATE) and hippocampal sclerosis.
2. **Interpretation**: Our findings suggest that cognitively defined AD subgroups may differ in select neuropathologic features, with evidence for differences in LATE and hippocampal sclerosis and less consistent patterns for AD neuropathologic change. These results support cognitive subtyping as a potentially useful framework for examining clinicopathologic heterogeneity, although substantial overlap across subgroups remains.
3. **Future directions**: Future studies are needed to replicate these findings in independent cohorts, incorporate quantitative and regionally resolved neuropathologic measures, and determine whether in vivo biomarkers can identify subgroup‐specific patterns of AD and co‐pathology during life.

## 2 METHODS

### 2.1 ACT study cohort

This study was approved by the Kaiser Permanente Interregional Institutional Review Board and the University of Washington (UW) Human Subjects Review Committees. The ACT study is a population‐based prospective cohort study focused on brain aging and risk factors for dementia that consists of community‐living members of Kaiser Permanente Washington (KPWA), formerly Group Health, and an integrated health‐care delivery organization in Washington state.^29^ A random sample of community‐living KPWA members aged ≥ 65 years without an established dementia diagnosis was invited to an enrollment visit between 1994 and 1996 (n =  2581). An expansion cohort (n = 811) was added between 2000 and 2003. In 2004, the study began ongoing enrollment to maintain a steady cohort size of 2000 to 2500 participants, enrolling new participants as participants leave the cohort due to death, dementia, or study discontinuation. Participants are invited to consider consenting for brain donation, but consent is not required to join the study; the brain donation consent rate is consistently 25% to 30%. Cognitive screening, physical function, medical history review, and functional status assessments are administered to ACT participants at study entry and subsequently every 2 years.

### 2.2 Cognitive assessments

Cognition is measured at each visit using the Cognitive Abilities Screening Instrument (CASI).^30^ The CASI assesses several cognitive domains, and scores range from 0 to 100, with higher scores indicating better cognition. Scores ≤ 85 prompt further clinical and psychometric evaluation, including a neuropsychological battery and a comprehensive neurological evaluation. The neuropsychological battery includes clock drawing,^31^ verbal fluency,^32^, ^33^ Mattis Dementia Rating Scale,^34^ Boston Naming Test,^33^, ^35^ verbal‐paired associations and recall, logical memory story immediate and delayed recall,^36^ CERAD Word List Memory,^33^, ^37^ Constructional Praxis and recall,^38^ Trails A and B,^39^ and Information and Comprehension subtest items.^8^ All cognitive and clinical data are reviewed in a multidisciplinary consensus conference to determine diagnoses of incident dementia and possible/probable AD using standardized (Diagnostic and Statistical Manual of Mental Disorders, Fourth Edition [DSM‐IV] and National Institute of Neurological and Communicative Disorders and Stroke and Alzheimer's Disease and Related Disorders Association [NINCDS‐ADRDA]) research criteria.^40^ Dementia‐free participants continue with biennial follow‐up.

### 2.3 Cognitive subgroups

We focus here on individuals who developed possible or probable AD dementia by NINCDS‐ADRDA criteria.^5^ As previously described, we obtained cognitive domain scores and used them to determine cognitively defined subgroups.^6^, ^8^, ^9^, ^10^, ^11^ Briefly, confirmatory factor analysis (CFA) was used to derive scores for four cognitive domains: memory, executive functioning, language, and visuospatial functioning. Next, an empirical relative‐domain thresholding approach was applied in which each participant's individual domain scores were expressed relative to the participant's average score across the four domains and compared to fixed cutoffs (e.g., −0.80 standard deviations^8^). Participants were then assigned to AD cognitive subgroups based on the number of domains substantially lower than their individual average (e.g., > 0.80 standard deviations below). Those with no such domains (i.e., all scores similar) were defined as having AD–no domain. Those with a single such domain were categorized as AD–memory, AD–executive, AD–language, or AD–visuospatial. Those with multiple such domains were classified as AD–multiple domains, but we excluded such participants from our analyses as too few died with brain donation (see Figure 1).

**FIGURE 1:**
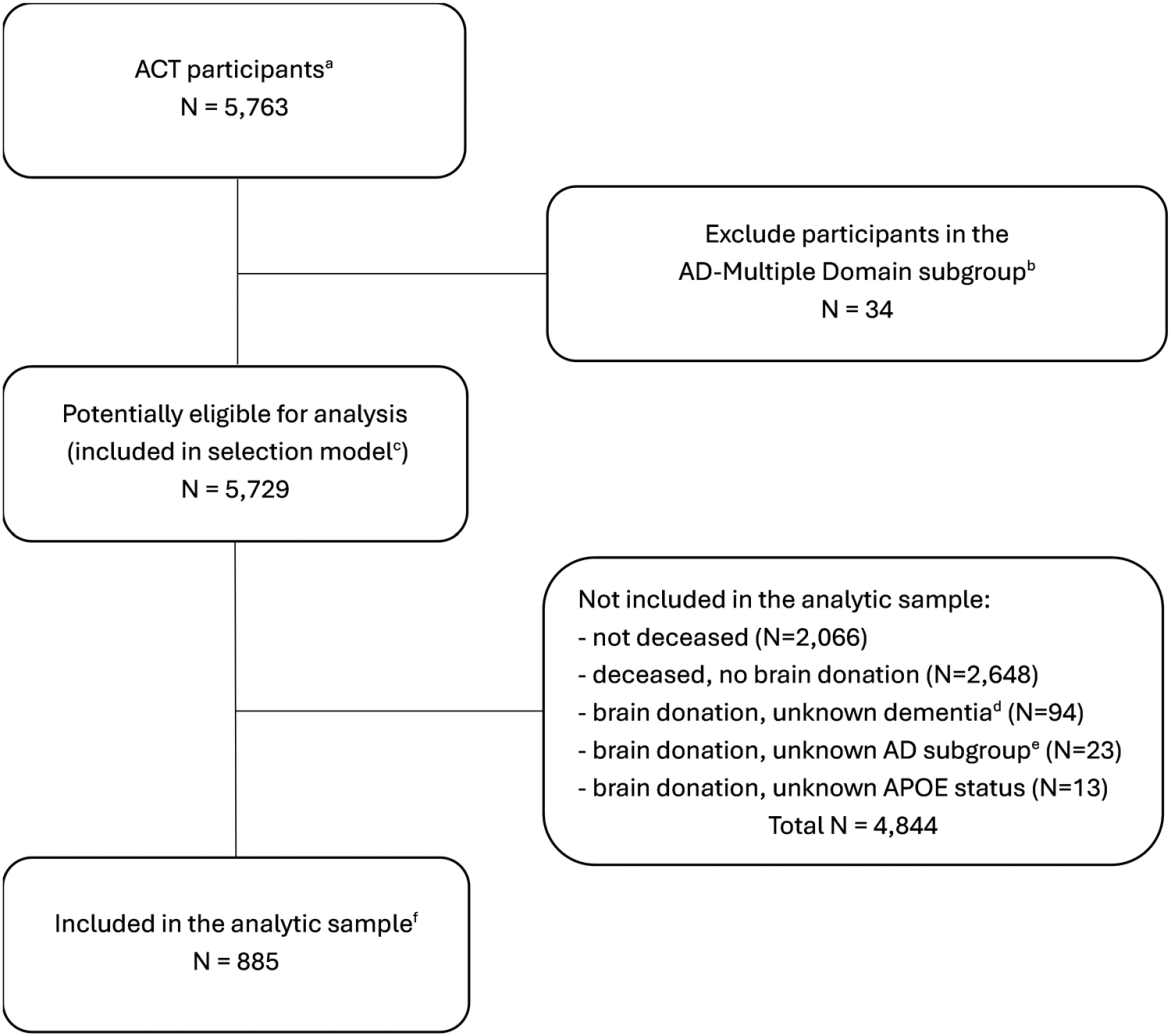
Flow diagram describing analytic study sample. ^a^Includes all participants ever enrolled in ACT as part of the study's original (1994–1996), expansion (2000–2003), or replacement (2004–2022) cohorts; longitudinal follow‐up data goes through March 31, 2024. ^b^Only 5 of these 34 died with brain donation, thus we excluded this group from analyses. ^c^Model for probability of inclusion in the analytic sample from this population of potentially eligible participants. ^d^Participants who had not been diagnosed with dementia in ACT but whose last cognitive evaluation was > 2 years prior to death. ^e^Participants who were diagnosed with possible/probable AD but had insufficient cognitive data available to assign them to an AD dementia cognitive subgroup. ^f^This is the overall analytic sample used in the article, but for any given neuropathology outcome analysis, the sample may be smaller due to missing data in the given outcome (see Table 2 for missing data by outcome). ACT, Adult Changes in Thought; AD, Alzheimer's disease; *APOE*, apolipoprotein E

### 2.4 Neuropathology assessments

Every ACT participant who provided written consent for research brain donation during life undergoes an extensive neuropathology evaluation by a board‐certified neuropathologist, as previously described.^41^, ^42^, ^43^ Briefly, all neuropathologic examinations are performed in the UW Division of Neuropathology and the UW BioRepository and Integrated Neuropathology (BRaIN) laboratory, blind to clinical diagnosis. For decedents with a *post mortem* interval < 12 hours, rapid brain procurement is performed, and tissue from one hemisphere is rapidly frozen. The remaining portion of the sampled hemisphere, and the unsampled (intact) contralateral hemisphere (or the whole brain in non‐rapid cases), are grossly examined and then immersion‐fixed in 10% neutral buffered formalin for at least 2 weeks.

After fixation, all brains are evaluated (wholly and after coronal sectioning) for any gross lesions, and atherosclerosis is assessed in the major arteries of the circle of Willis. Tissue samples are dissected from middle frontal gyrus, superior and middle temporal gyri, inferior parietal lobule, anterior cingulate gyrus, primary visual cortex, basal ganglia at the level of the anterior commissure, thalamus, hippocampus at the level of the uncus and lateral geniculate nucleus, amygdala, midbrain including substantia nigra, pons at the level of the locus coeruleus, medulla, and cerebellar hemisphere. All these samples are processed and embedded in paraffin prior to sectioning and staining. A microtome is used to cut 4 µm‐thick tissue sections from formalin fixed paraffin embedded (FFPE) tissue blocks which are stained with hematoxylin and eosin (H&E) and Luxol fast blue (LFB). A Bielschowsky silver stain is manually performed on 8 µm‐thick tissue sections of mid‐frontal and parietal sections.

H&E/LFB‐stained slides are used to evaluate arteriolosclerosis and to identify chronic gross and microinfarcts. Immunohistochemistry is performed on multiple sections to assess neuropathology according to current consensus guidelines, including AD neuropathologic change (ADNC), defined by Thal phase, Braak stage, and CERAD score.^15^ Additional assessments include cerebral amyloid angiopathy (CAA), Lewy body disease (LBD) pathology, LATE neuropathologic change (LATE‐NC), and hippocampal sclerosis (HS).^15^, ^23^, ^27^, ^44^ As these metrics have evolved, we have conducted retrospective assessments, as needed, of all historical cases with available tissue. Appropriate positive controls are included for each antibody; negative controls are also run, consisting of secondary antibodies of the appropriate species in the absence of primary antibody.

Each neuropathologic outcome was defined and graded using established consensus criteria, and all ordinal measures were analyzed at their full ordinal resolution without collapsing categories. The overall ADNC score was classified as none, low, intermediate, or high per NIA‐AA ABC scoring guidelines.^15^ Its component measures were retained as separate ordinal outcomes: Thal phase (0–5), reflecting the hierarchical spread of Aβ deposits across the brain; Braak stage (0, I, II, III, IV, V, or VI), reflecting the extent of NFT pathology; and CERAD neuritic plaque score (absent, sparse, moderate, or frequent). CAA was graded on a 0 to 3 scale per lobe using the Love et al. consensus protocol,^44^ and the maximum score across sampled cortical lobes was used as the analytic variable. LATE‐NC was staged 0 to 3 per Nelson et al. consensus criteria:^27^ stage 0, no TDP‐43 pathology detected; stage 1, TDP‐43 pathology restricted to the amygdala; stage 2, additional involvement of the hippocampus and/or entorhinal cortex; stage 3, additional involvement of the middle frontal gyrus. HS was recorded as present or absent. LBD pathology was classified as absent or present, with subtype noted as brainstem predominant, amygdala predominant, limbic (transitional), or neocortical per McKeith et al.;^23^ we used this information to construct an LBD outcome with four levels (absent, brainstem predominant, limbic or amygdala predominant, and neocortical) which we treated as an approximate ordinal variable (absent → more diffuse) for the purposes of our investigations. For vascular outcomes, arteriolosclerosis was graded as absent, mild, moderate, or severe; atherosclerosis of the circle of Willis was graded on a 0 to 3 scale; and the counts of chronic gross infarcts and microinfarcts (reported overall and separately for cerebral cortical and deep screening sections) were each recorded as 0, 1, 2, or 3+.

For this article, we organize our analyses and results by grouping neuropathology outcomes into three separate domains: ADNC measures (ADNC score, Thal phase, Braak stage, CERAD score, and CAA); non‐ADNC, non‐vascular pathology measures (LATE‐NC, HS, and LBD); and vascular pathology measures (gross infarcts, microinfarcts [any, cerebral, deep], atherosclerosis, and arteriolosclerosis).

### 2.5 Covariates

Covariates are provided as of the ACT biennial study visit at which participants were diagnosed with dementia or from the last study visit for those who died without dementia. Participant demographic characteristics included age; years of ACT biennial visit follow‐up; and self‐reported sex, race/ethnicity, and years of education. Apolipoprotein E (*APOE*) genotype information was also available. Cognitive status was either AD dementia (i.e., possible/probable AD per NINCDS‐ADRDA criteria), non‐AD dementia (i.e., dementia per DSM‐IV but without meeting possible/probable AD criteria), or no dementia. Participants with AD dementia were further categorized into the subgroups of interest (AD–no domain, AD–memory, AD–visuospatial, AD–language, and AD–executive) as described in section 2.3. Other health and functional characteristics that were used in selection models (described in section 2.6) included: self‐reported histories of coronary artery disease and cerebrovascular disease; the number of activities of daily living (ADL)—walking around the house, getting out of bed or a chair, getting to or using the toilet, and feeding, dressing, and showering themselves with which they reported any difficulty; and a study‐assessed measure of a 10‐foot timed walk task.

### 2.6 Statistical analysis

For each neuropathology outcome, we fit an ordinal logistic regression model with a 7‐level categorical predictor indicating each participant's group membership—AD–no domain, AD–memory, AD–visuospatial, AD–language, AD–executive, non‐AD dementia, or no dementia—to estimate associations with the odds of higher levels of neuropathology. Model estimates were adjusted for sex and the continuous variables of age at death, years of education, and the number of years after dementia diagnosis that the participant lived. Each of these continuous variables was parameterized using restricted cubic splines with two degrees of freedom. Estimates were additionally adjusted for *APOE* genotype, categorized as at least one ε4 allele versus no ε4 allele, though we also provide supplemental results without *APOE* adjustment for comparison. Because the generalizability of analyses performed in a brain donation sample may be affected by selection mechanisms related to consent to brain donation, death, or study withdrawal, we also adjusted our ordinal logistic regression models using inverse probability weighting (IPW).^45^ The weights were derived by estimating among all potentially eligible participants (see Figure 1) the probability of inclusion in the analytic sample as a function of: dementia status (no dementia, AD dementia, or non‐AD dementia); sex; age (at dementia diagnosis or latest visit); years of education; time on ACT study (modeled using restricted cubic splines as before); the health and functional measures described previously in section 2.5; and interactions between dementia status and all covariates.

We used the adjusted and weighted ordinal logistic regression models to estimate odds ratios (ORs) with 95% confidence intervals (CIs) for each neuropathology outcome. These estimates correspond to the seven‐level categorical exposure, using participants without dementia as the reference group. Further, to facilitate comparisons among the five AD‐dementia cognitive subgroups specifically, from this same resultant model we additionally provide plots of estimated ORs and 95% CIs for each of the AD–memory, AD–visuospatial, AD–language, and AD–executive groups relative to the AD–no domain group. *P* values corresponding to omnibus Wald tests for jointly assessing multiple contrasts at once are provided. As we examined multiple outcome measures within each of three separate neuropathology domains (i.e., ADNC: five outcome measures; non‐AD, non‐vascular pathology: three outcome measures; and vascular pathology: six outcome measures), we indicate which *P* values remain significant (using the original α = 0.05 level) after applying Holm–Bonferroni adjustments separately within each of those three neuropathology domains.^46^ Subgroup‐specific estimates are interpreted descriptively. A non‐significant omnibus *P* value does not preclude meaningful associations between individual subgroups and a given neuropathology outcome; patterns observed in the context of a non‐significant omnibus test are therefore interpreted descriptively and framed as hypothesis generating. All analyses were conducted using R, version 4.3.3,^47^ with regression model estimation and selection weight incorporation achieved using the svyVGAM package, version 1.2.^48^, ^49^

## 3 RESULTS

Our analyses used data from the ACT study's 2024 data release, which included study visit and *post mortem* neuropathology information for participants through March 31, 2024. Figure 1 illustrates the criteria applied to arrive at the final analytic sample used in the analyses in this article. Among 5729 ACT participants, 885 (15%) died, donated their brains for neuropathology evaluation, and had the required dementia and AD subgroup status and *APOE* genotype information needed for our analyses. As noted in the figure, participants in the included sample who had not been diagnosed with dementia in ACT prior to death were required to have had an ACT visit within the 2 years preceding death to ensure a recent assessment of cognition.

### 3.1 Demographic characteristics of the analytic cohort

Table 1 presents participant characteristics overall and stratified by dementia and AD subgroup status. The prevalence of each of the dementia groups in this sample was: no dementia (49%), non‐AD dementia (9%), and AD dementia (42%), with the latter including AD–no domain (23%), AD–memory (6%), AD–visuospatial (6%), AD–language (4%), and AD–executive (3%). Women constituted 58% of the sample overall, with higher proportions among those with AD dementia (> 60% women in each AD subgroup, except the AD–visuospatial group). Participants reported an average of 15 years of education, with averages ranging from 13 to 15 years across subgroups. The average age at AD diagnosis was 86 to 87 across subgroups, and the average time from AD diagnosis to death was ≈ 4 to 5 years. A comparison of characteristics between the analytic cohort and the broader sample of ACT participants from which they were derived is provided in Table S1 in supporting information.

**TABLE 1:** Participant characteristics, overall and stratified by dementia and AD dementia cognitive subgroup classification.

				AD dementia^c^				
Characteristics	Total (*n* = 885)	No dementia^a^ (*n* = 438)	Non‐AD dementia^b^ (*n* = 77)	AD–no domain (*n* = 201)	AD–memory (*n* = 56)	AD‐ –visuospatial (*n* = 55)	AD–language (*n* = 31)	AD–executive (*n* = 27)
Women	512 (58%)	238 (54%)	38 (49%)	129 (64%)	39 (70%)	31 (56%)	20 (65%)	17 (63%)
Non‐Hispanic White^d^	828 (94%)	415 (95%)	75 (97%)	186 (93%)	49 (88%)	52 (95%)	29 (94%)	22 (81%)
Years of biennial visit follow‐up	10.1 (6.0)	10.5 (6.3)	9.2 (5.8)	10.3 (5.9)	9.9 (6.0)	9.5 (5.7)	9.2 (5.2)	9.3 (4.9)
Age at visit	86.3 (6.5)	86.4 (7.0)	84.6 (5.9)	86.5 (6.0)	86.8 (6.3)	86.3 (5.8)	86.6 (6.2)	86.8 (6.2)
Years from last assessment to death	2.7 (2.8)	1.0 (0.6)	3.4 (2.6)	4.8 (3.3)	4.4 (2.8)	4.8 (4.0)	4.1 (2.2)	4.3 (2.7)
Years of education	14.9 (3.0)	15.2 (3.0)	15.0 (3.5)	14.3 (2.8)	14.5 (3.0)	15.2 (3.2)	15.3 (2.5)	13.3 (3.3)
At least one *APOE* ε4 allele	256 (29%)	103 (24%)	30 (39%)	71 (35%)	25 (45%)	15 (27%)	8 (26%)	4 (15%)
Coronary artery disease	272 (31%)	156 (36%)	23 (30%)	48 (24%)	12 (21%)	18 (33%)	9 (29%)	6 (22%)
Cerebrovascular disease	258 (29%)	131 (30%)	38 (49%)	43 (21%)	10 (18%)	19 (35%)	11 (35%)	6 (22%)
# of ADLs with difficulty^e^	1.5 (1.8)	1.6 (1.8)	2.4 (2.1)	1.1 (1.5)	0.7 (1.2)	1.7 (1.8)	1.0 (1.6)	1.9 (1.9)
Unable to perform timed walking task^f^	45 (7%)	26 (8%)	4 (10%)	8 (5%)	1 (2%)	4 (9%)	0 (0%)	2 (11%)

*Note*: Data provided in this table are from the biennial study visit at which participants were diagnosed with dementia (of any type) or from the last study visit for those who died without dementia (cognitively normal controls). Values shown in this table are either n (%) or mean (standard deviation) depending on the characteristic.

Abbreviations: AD, Alzheimer's disease; ADL, activity of daily living; *APOE*, apolipoprotein E; DSM‐IV, Diagnostic and Statistical Manual of Mental Disorders, Fourth Edition; NINCDS‐ADRDA, National Institute of Neurological and Communicative Disorders and Stroke and Alzheimer's Disease and Related Disorders Association.

^a^ Participants not diagnosed with dementia or AD.

^b^ Participants diagnosed with dementia per DSM‐IV criteria but who did not meet NINCDS‐ADRDA criteria for AD.

^c^ Participants who met NINCDS‐ADRDA criteria for AD.

^d^ Due to small proportions of participants in most race/ethnicity categories, we only report the proportion of participants categorized as non‐Hispanic White, which was the most common category.

^e^ ADL information was unavailable for 74 (8%) of participants.

^f^ The 10‐foot timed walking task was not assessed for 225 (25%) of participants; therefore, % unable calculated among those assessed.

### 3.2 Cognitive domain scores of the analytic cohort

Table 2 provides summaries of the distribution of cognitive domain scores (*z* scores) in participants without dementia and those diagnosed with non‐AD dementia or AD dementia, with the latter group broken out by AD cognitive subgroup. As expected, participants with AD dementia demonstrated markedly lower cognitive scores, on average, in the specific domains which defined their respective AD subgroups. The distribution of scores in the AD–no domain was more comparable to the distribution in the non‐AD dementia group.

**TABLE 2:** Cognitive domain scores stratified by dementia and AD dementia cognitive subgroup classification.

			AD dementia^c^				
Cognitive domain score	No dementia^a^ (*n* = 438)	Non‐AD dementia^b^ (*n* = 77)	AD–no domain (*n* = 201)	AD–memory (*n* = 56)	AD–visuospatial (*n* = 55)	AD–language (*n* = 31)	AD–executive (*n* = 27)
Memory	0.20 (0.60)	−0.78 (0.42)	−0.80 (0.34)	−1.32 (0.38)	−0.61 (0.30)	−0.76 (0.55)	−0.67 (0.59)
Visuospatial functioning	0.04 (0.51)	−0.35 (0.78)	−0.24 (0.60)	0.37 (0.71)	−1.14 (0.64)	−0.02 (0.82)	−0.25 (0.68)
Language	0.14 (0.62)	−0.73 (0.64)	−0.57 (0.43)	−0.40 (0.56)	−0.26 (0.51)	−1.42 (0.75)	−0.75 (0.50)
Executive functioning	0.03 (0.67)	−1.03 (0.73)	−0.63 (0.52)	−0.30 (0.53)	−0.40 (0.42)	−0.94 (0.85)	−1.69 (0.59)

*Note*: Values shown in this table are mean (standard deviation) of the cognitive domain scores (*z* scores).

Abbreviations: AD, Alzheimer's disease; DSM‐IV, Diagnostic and Statistical Manual of Mental Disorders, Fourth Edition; NINCDS‐ADRDA, National Institute of Neurological and Communicative Disorders and Stroke and Alzheimer's Disease and Related Disorders Association.

^a^ Participants not diagnosed with dementia or AD. Some cognitive domain scores were missing for these participants: memory, *n* = 38 (9%); visuospatial functioning, *n* = 50 (11%); language, *n* = 39 (9%); and executive functioning, *n* = 38 (9%).

^b^ Participants diagnosed with dementia per DSM‐IV criteria but who did not meet NINCDS‐ADRDA criteria for AD. Some cognitive domain scores were missing for these participants: memory, *n* = 4 (5%); visuospatial functioning, *n* = 13 (17%); language, *n* = 4 (5%); and executive functioning, *n* = 6 (8%).

^c^ Participants who met NINCDS‐ADRDA criteria for AD. No cognitive domain score were missing for these participants (by design).

### 3.3 Neuropathologic features of the analytic cohort

Table 3 provides a comprehensive summary of the distribution of each neuropathology outcome overall and across groups. Although many individuals without dementia harbor some degree of pathology, the unadjusted comparisons of distributions of ADNC measures illustrated in this table show higher proportions of more severe levels of pathology (e.g., Thal phase 4/5, Braak stage V/VI, CERAD score “frequent”) in participants with AD dementia compared to those without. Additionally, despite lacking a clinical diagnosis of AD dementia, the non‐AD dementia group had significant AD‐related pathology, with most individuals (68%) having intermediate or high ADNC score.

**TABLE 3:** Neuropathology outcomes, overall and stratified by dementia and AD dementia cognitive subgroup classification.

				AD dementia				
Neuropathology outcomes	Total (*n* = 885)	No dementia (*n* = 438)	Non‐AD dementia (*n* = 77)	AD–no domain (*n* = 201)	AD–memory (*n* = 56)	AD–visuospatial (*n* = 55)	AD–language (*n* = 31)	AD–executive (*n* = 27)
**ADNC measures**								
ADNC score^a^								
None	95 (11%)	68 (16%)	3 (4%)	13 (6%)	2 (4%)	6 (11%)	2 (6%)	1 (4%)
Low	250 (29%)	184 (43%)	20 (28%)	23 (12%)	7 (13%)	9 (17%)	4 (13%)	3 (11%)
Intermediate	261 (30%)	127 (30%)	25 (35%)	66 (33%)	9 (17%)	16 (30%)	10 (32%)	8 (30%)
High	262 (30%)	51 (12%)	24 (33%)	98 (49%)	36 (67%)	23 (43%)	15 (48%)	15 (56%)
Thal phase^b^								
0	97 (11%)	69 (16%)	4 (5%)	13 (6%)	2 (4%)	6 (11%)	2 (6%)	1 (4%)
1	103 (12%)	73 (17%)	11 (15%)	10 (5%)	5 (9%)	2 (4%)	0 (0%)	2 (7%)
2	82 (9%)	52 (12%)	4 (5%)	10 (5%)	3 (5%)	9 (16%)	3 (10%)	1 (4%)
3	158 (18%)	87 (20%)	13 (18%)	33 (16%)	6 (11%)	9 (16%)	6 (19%)	4 (15%)
4	253 (29%)	115 (27%)	26 (35%)	63 (31%)	12 (21%)	18 (33%)	10 (32%)	9 (33%)
5	183 (21%)	36 (8%)	16 (22%)	72 (36%)	28 (50%)	11 (20%)	10 (32%)	10 (37%)
Braak stage^c^								
0	23 (3%)	19 (4%)	0 (0%)	2 (1%)	1 (2%)	0 (0%)	0 (0%)	1 (4%)
I	62 (7%)	51 (12%)	3 (4%)	5 (2%)	1 (2%)	1 (2%)	1 (3%)	0 (0%)
II	129 (15%)	97 (22%)	11 (15%)	12 (6%)	0 (0%)	7 (13%)	1 (3%)	1 (4%)
III	134 (15%)	91 (21%)	11 (15%)	17 (8%)	1 (2%)	7 (13%)	5 (16%)	2 (7%)
IV	171 (19%)	98 (22%)	14 (19%)	35 (18%)	8 (15%)	8 (15%)	4 (13%)	4 (15%)
V	216 (25%)	66 (15%)	25 (33%)	67 (34%)	21 (39%)	17 (31%)	10 (32%)	10 (37%)
VI	142 (16%)	14 (3%)	11 (15%)	62 (31%)	22 (41%)	14 (26%)	10 (32%)	9 (33%)
CERAD score								
Absent	205 (23%)	151 (34%)	7 (9%)	25 (12%)	7 (12%)	10 (18%)	2 (6%)	3 (11%)
Sparse	183 (21%)	111 (25%)	25 (32%)	28 (14%)	4 (7%)	5 (9%)	4 (13%)	6 (22%)
Moderate	229 (26%)	105 (24%)	20 (26%)	49 (24%)	16 (29%)	24 (44%)	9 (29%)	6 (22%)
Frequent	268 (30%)	71 (16%)	25 (32%)	99 (49%)	29 (52%)	16 (29%)	16 (52%)	12 (44%)
CAA								
0	404 (46%)	231 (53%)	31 (40%)	81 (40%)	15 (27%)	26 (47%)	13 (42%)	7 (26%)
1	176 (20%)	86 (20%)	14 (18%)	36 (18%)	13 (23%)	14 (25%)	7 (23%)	6 (22%)
2	278 (31%)	114 (26%)	31 (40%)	75 (37%)	27 (48%)	12 (22%)	7 (23%)	12 (44%)
3	27 (3%)	7 (2%)	1 (1%)	9 (4%)	1 (2%)	3 (5%)	4 (13%)	2 (7%)
**Non‐ADNC, non‐vascular pathology**								
LATE‐NC^d^								
Stage 0	435 (50%)	266 (62%)	41 (57%)	73 (36%)	16 (29%)	19 (35%)	9 (29%)	11 (41%)
Stage 1	186 (21%)	93 (22%)	11 (15%)	46 (23%)	7 (12%)	19 (35%)	5 (16%)	5 (19%)
Stage 2	222 (26%)	64 (15%)	18 (25%)	70 (35%)	29 (52%)	16 (30%)	15 (48%)	10 (37%)
Stage 3	23 (3%)	3 (1%)	2 (3%)	11 (6%)	4 (7%)	0 (0%)	2 (6%)	1 (4%)
Hippocampal sclerosis present^e^	139 (16%)	30 (7%)	11 (14%)	47 (24%)	24 (45%)	11 (20%)	9 (29%)	7 (26%)
Lewy body disease^f^								
Absent	678 (77%)	363 (83%)	56 (73%)	151 (75%)	35 (62%)	33 (60%)	21 (68%)	19 (70%)
Brainstem‐predominant	26 (3%)	14 (3%)	1 (1%)	4 (2%)	2 (4%)	3 (5%)	2 (6%)	0 (0%)
Limbic or amygdala‐predominant	103 (12%)	36 (8%)	9 (12%)	31 (15%)	14 (25%)	8 (15%)	1 (3%)	4 (15%)
Neocortical	77 (9%)	24 (5%)	11 (14%)	15 (7%)	5 (9%)	11 (20%)	7 (23%)	4 (15%)
**Vascular pathology**								
# of gross infarcts^g^								
0	585 (66%)	332 (76%)	39 (51%)	118 (59%)	37 (66%)	32 (58%)	11 (35%)	16 (59%)
1	128 (14%)	48 (11%)	14 (18%)	33 (16%)	12 (21%)	9 (16%)	6 (19%)	6 (22%)
2	77 (9%)	27 (6%)	11 (14%)	23 (12%)	3 (5%)	6 (11%)	6 (19%)	1 (4%)
3+	93 (11%)	31 (7%)	12 (16%)	26 (13%)	4 (7%)	8 (15%)	8 (26%)	4 (15%)
# of microinfarcts, any location^h^								
0	465 (53%)	254 (58%)	37 (48%)	96 (48%)	33 (60%)	24 (44%)	11 (35%)	10 (37%)
1	177 (20%)	87 (20%)	8 (10%)	40 (20%)	11 (20%)	17 (31%)	10 (32%)	4 (15%)
2	93 (11%)	41 (9%)	8 (10%)	28 (14%)	4 (7%)	4 (7%)	3 (10%)	5 (19%)
3+	146 (17%)	54 (12%)	24 (31%)	37 (18%)	7 (13%)	9 (17%)	7 (23%)	8 (30%)
# of microinfarcts, cerebral cortical^h^								
0	578 (66%)	306 (70%)	46 (60%)	127 (63%)	38 (69%)	31 (57%)	16 (52%)	14 (52%)
1	139 (16%)	62 (14%)	7 (9%)	33 (16%)	7 (13%)	15 (28%)	8 (26%)	7 (26%)
2	78 (9%)	37 (8%)	7 (9%)	17 (8%)	5 (9%)	5 (9%)	3 (10%)	4 (15%)
3+	86 (10%)	31 (7%)	17 (22%)	24 (12%)	5 (9%)	3 (6%)	4 (13%)	2 (7%)
# of microinfarcts, deep^h^								
0	636 (72%)	341 (78%)	48 (62%)	131 (65%)	44 (80%)	37 (69%)	19 (61%)	16 (59%)
1	143 (16%)	56 (13%)	12 (16%)	46 (23%)	8 (15%)	9 (17%)	6 (19%)	6 (22%)
2	65 (7%)	26 (6%)	11 (14%)	18 (9%)	2 (4%)	3 (6%)	4 (13%)	1 (4%)
3+	37 (4%)	13 (3%)	6 (8%)	6 (3%)	1 (2%)	5 (9%)	2 (6%)	4 (15%)
Atherosclerosis^i^								
0	31 (4%)	25 (6%)	0 (0%)	5 (2%)	1 (2%)	0 (0%)	0 (0%)	0 (0%)
1	199 (23%)	122 (28%)	18 (24%)	25 (12%)	11 (20%)	10 (19%)	5 (17%)	8 (31%)
2	560 (64%)	262 (61%)	43 (57%)	151 (75%)	34 (62%)	39 (72%)	18 (60%)	13 (50%)
3	82 (9%)	21 (5%)	15 (20%)	20 (10%)	9 (16%)	5 (9%)	7 (23%)	5 (19%)
Arteriolosclerosis^j^								
Absent	7 (1%)	5 (1%)	0 (0%)	1 (1%)	0 (0%)	0 (0%)	1 (4%)	0 (0%)
Mild	171 (22%)	106 (29%)	10 (16%)	29 (15%)	9 (17%)	12 (22%)	1 (4%)	4 (17%)
Moderate	398 (51%)	193 (53%)	29 (45%)	104 (55%)	26 (50%)	20 (37%)	16 (57%)	10 (43%)
Severe	197 (25%)	58 (16%)	25 (39%)	56 (29%)	17 (33%)	22 (41%)	10 (36%)	9 (39%)

Abbreviations: AD, Alzheimer's disease; ADNC, Alzheimer's disease neuropathologic change; CAA, cerebral amyloid angiopathy; CERAD, Consortium to Establish a Registry for Alzheimer's Disease; LATE‐NC, limbic‐predominant age‐related transactive response DNA binding protein 43 kDa encephalopathy neuropathologic change.

^a^ ADNC score was unavailable for 17 (2%) of participants.

^b^ Thal phase was unavailable for 9 (1%) of participants.

^c^ Braak stage was unavailable for 8 (1%) of participants.

^d^ LATE‐NC was unavailable for 19 (2%) of participants.

^e^ Hippocampal sclerosis was unavailable for 19 (2%) of participants.

^f^ Lewy body disease was unavailable for 1 (0.1%) of participants.

^g^ Gross infarcts were unavailable for 2 (0.2%) of participants.

^h^ Microinfarcts were unavailable for 4 (0.5%) of participants.

^i^ Atherosclerosis was unavailable for 13 (1%) of participants.

^j^ Arteriolosclerosis was unavailable for 112 (13%) of participants.

### 3.4 Neuropathology outcomes in dementia versus non‐dementia controls

The primary model estimates comparing pathology levels in the AD dementia subgroups and the non‐AD dementia group with those in the no dementia group are provided in Table 4 (with adjustment for *APOE*) and Table S2 in supporting information (without adjustment for *APOE*). As expected, the AD dementia subgroups had higher odds of more severe pathology as measured by Thal phase, Braak stage, and CERAD score (and the combined ADNC score) compared to those without dementia; *P* values corresponding to five degrees of freedom (df) omnibus tests of any differences between the five AD dementia subgroups and the no dementia group were ≤ 0.002 for each of these outcomes (regardless of whether adjusting for *APOE*). The non‐AD dementia group also had higher estimated odds for most of these outcomes compared to those without dementia, but the point estimates tended to be of smaller magnitude than those estimated for the AD dementia subgroups. We did not observe significant differences for CAA. For the non‐ADNC, non‐vascular neuropathologic outcomes, estimates suggested evidence of more severe pathology in the AD‐dementia subgroups relative to the no dementia group: LATE‐NC (*P* = 0.002), HS (*P* < 0.001), and LBD (*P* = 0.017), with similar results in models not adjusted for *APOE*. We did not detect significant differences in these outcomes, however, between participants with non‐AD dementia and participants without dementia. Conversely, for the vascular neuropathology outcomes, the opposite tended to be suggested. We did not detect significant differences in estimated odds of more severe vascular pathology in the AD‐dementia subgroups relative to participants without dementia, with the exception of gross infarcts (*P* = 0.004 and 0.003, with and without *APOE* adjustment, respectively); whereas estimates for those with non‐AD dementia were consistently greater than those without dementia for the vascular measures.

**TABLE 4:** Comparisons of neuropathology levels: (1) between participants with non‐AD dementia and participants without dementia; and (2) between AD dementia cognitive subgroups and participants without dementia.

		Each AD dementia cognitive subgroup vs. No dementia					
Neuropathology	Non‐AD dementia vs. No dementia OR (95% CI)^a^	AD–no domain OR (95% CI)	AD–memory OR (95% CI)	AD–visuospatial OR (95% CI)	AD–language OR (95% CI)	AD–executive OR (95% CI)	Omnibus *P* value^b^
**ADNC measures**							
ADNC score	**2.38 (1.38, 4.08)**	**3.63 (2.23, 5.92)**	**7.86 (3.74, 16.50)**	**2.97 (1.50, 5.88)**	**5.46 (2.41, 12.38)**	**5.45 (1.98, 15.01)**	<0.001*
Thal phase	1.62 (0.92, 2.84)	**2.29 (1.39, 3.77)**	**3.75 (1.84, 7.64)**	1.33 (0.73, 2.43)	**2.77 (1.22, 6.25)**	2.59 (0.98, 6.86)	0.002*
Braak stage	**2.69 (1.69, 4.28)**	**4.21 (2.70, 6.57)**	**9.47 (4.83, 18.56)**	**2.72 (1.45, 5.11)**	**6.31 (2.88, 13.80)**	**5.72 (2.38, 13.76)**	<0.001*
CERAD score	**2.07 (1.19, 3.60)**	**2.93 (1.83, 4.69)**	**3.61 (1.89, 6.90)**	1.86 (0.98, 3.55)	**4.61 (2.18, 9.74)**	**2.89 (1.25, 6.69)**	<0.001*
CAA	0.94 (0.54, 1.62)	1.00 (0.62, 1.61)	1.51 (0.84, 2.72)	0.73 (0.38, 1.38)	1.25 (0.52, 3.01)	2.26 (0.91, 5.57)	0.191
**Non‐ADNC, non‐vascular pathology**							
LATE‐NC	0.92 (0.53, 1.60)	**1.72 (1.06, 2.78)**	**3.23 (1.65, 6.29)**	1.19 (0.65, 2.18)	**4.07 (1.70, 9.76)**	1.70 (0.75, 3.84)	0.002*
Hippocampal sclerosis	1.94 (0.78, 4.81)	**2.41 (1.18, 4.91)**	**7.49 (3.33, 16.85)**	1.68 (0.67, 4.24)	**5.29 (1.79, 15.58)**	2.66 (0.87, 8.13)	<0.001*
Lewy body disease	1.78 (0.96, 3.31)	1.37 (0.76, 2.49)	**2.58 (1.24, 5.36)**	**2.69 (1.33, 5.45)**	**2.61 (1.01, 6.75)**	2.41 (0.85, 6.83)	0.017*
**Vascular pathology**							
Gross infarcts	**3.72 (2.18, 6.35)**	**2.24 (1.35, 3.70)**	1.43 (0.73, 2.80)	**2.24 (1.16, 4.30)**	**4.23 (1.97, 9.09)**	2.18 (0.88, 5.41)	0.004*
Any microinfarcts	**2.50 (1.40, 4.48)**	1.64 (1.03, 2.61)	1.03 (0.54, 1.96)	1.52 (0.85, 2.73)	2.11 (0.99, 4.51)	2.78 (1.24, 6.21)	0.081
Cerebral microinfarcts	**2.26 (1.30, 3.94)**	1.38 (0.83, 2.28)	1.11 (0.56, 2.21)	1.45 (0.77, 2.72)	2.04 (0.90, 4.60)	1.72 (0.76, 3.90)	0.547
Deep microinfarcts	**2.87 (1.50, 5.49)**	1.99 (1.16, 3.41)	0.97 (0.45, 2.11)	1.73 (0.84, 3.55)	2.21 (0.92, 5.34)	2.96 (1.17, 7.50)	0.054
Atherosclerosis	**1.89 (1.01, 3.54)**	1.20 (0.73, 1.98)	1.42 (0.69, 2.93)	1.04 (0.56, 1.93)	1.84 (0.60, 5.58)	0.97 (0.34, 2.71)	0.842
Arteriolosclerosis	**2.87 (1.49, 5.51)**	1.33 (0.78, 2.28)	1.49 (0.74, 3.01)	1.76 (0.86, 3.60)	1.68 (0.73, 3.89)	1.97 (0.63, 6.12)	0.592

*Note*: OR and 95% CI are estimated from ordinal logistic regression models—a separate model for each neuropathology outcome—adjusted for sex, age at death, years of education, years from visit to death, and *APOE*, and incorporating inverse‐probability weighting to account for selection into the analytic sample. The reference group for all estimates shown in this table is the No dementia group. ORs > 1 correspond to higher estimated levels of the neuropathology outcome in the specified group (relative to No dementia).

Abbreviations: AD, Alzheimer's disease; ADNC, Alzheimer's disease neuropathologic change; *APOE*, apolipoprotein E; CAA, cerebral amyloid angiopathy; CERAD, Consortium to Establish a Registry for Alzheimer's Disease; CI, confidence interval; df, degrees of freedom; LATE‐NC, limbic‐predominant age‐related transactive response DNA binding protein 43 kDa encephalopathy neuropathologic change; OR, odds ratio.

^a^ For the Non‐AD dementia versus No dementia comparisons, estimates for which the 95% CI excludes 1 are in bold.

^b^ *P* values correspond to 5‐degrees of freedom omnibus tests of any significant differences in estimated odds between the 5 AD dementia subgroups and the No dementia group for the specified neuropathology outcome. While the reported *P* values have not been adjusted for multiple comparisons (5 AD models, 3 non‐AD/non‐vascular models, and 6 vascular models), we have denoted with an asterisk (*) those that retain significance (original α = 0.05) after applying Holm–Bonferroni adjustments separately per neuropathology domain; only in those instances do we additionally **bold** any corresponding AD dementia subgroup pairwise estimates for which the original 95% CI excludes 1.

### 3.5 Neuropathology outcomes across AD dementia subgroups

Primary model estimated ORs and 95% CIs comparing neuropathology levels across the AD dementia subgroups are provided in Figure 2 (for ADNC measures), Figure 3 (for non‐ADNC, non‐vascular pathology), and Figure 4 (for vascular pathology). For comparison, results from models without adjustment for *APOE* are provided in Figures S1–S3 in supporting information.

**FIGURE 2:**
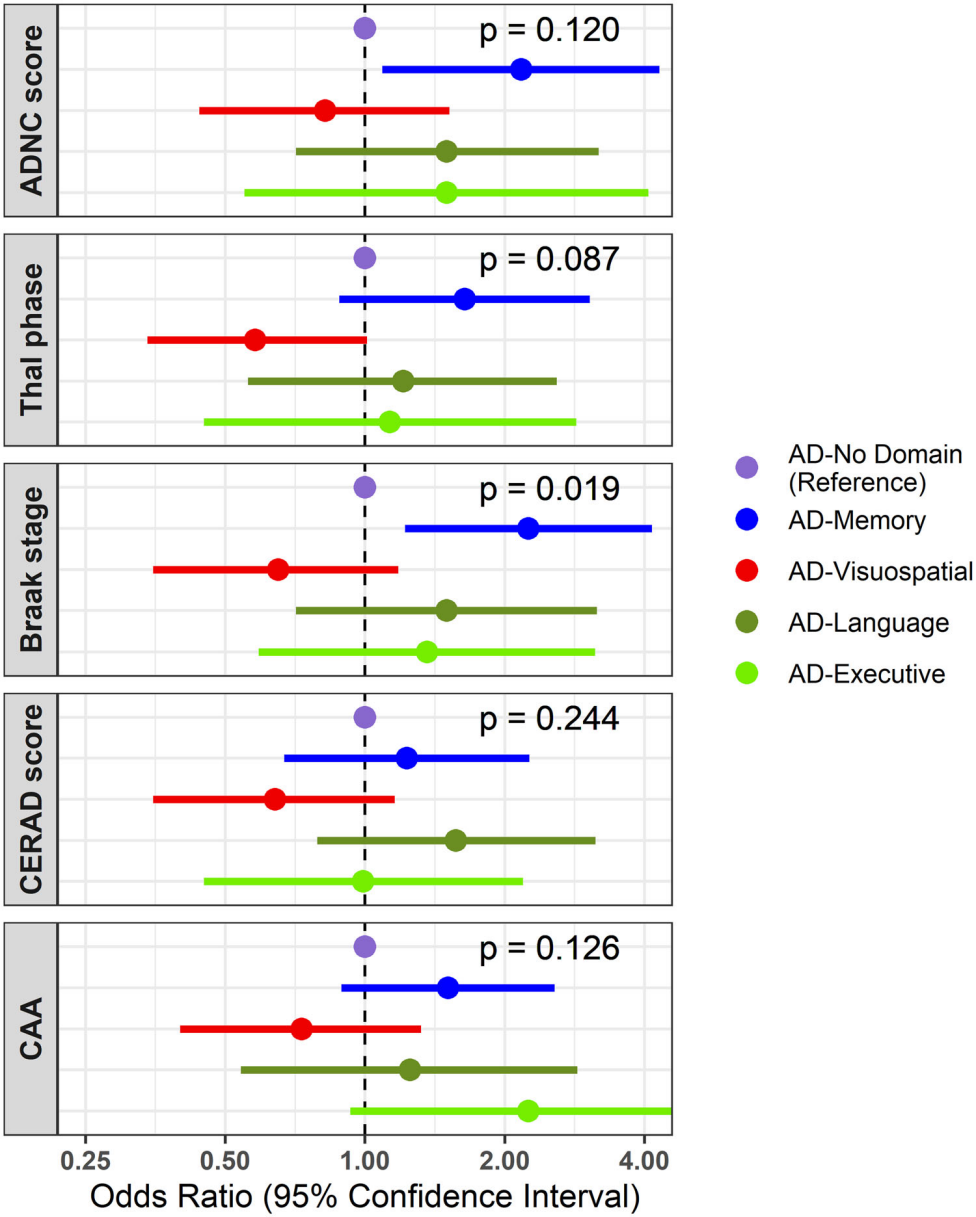
Comparisons of ADNC levels across the AD dementia cognitive subgroups. OR and 95% CI are estimated from the same models used to produce Table 4 results, but results here are focused on the comparisons between AD dementia subgroups. OR axis is shown using a log scale. The reference group for all estimates shown in this figure is the AD–no domain dementia subgroup. *P* values correspond to 4‐degrees of freedom omnibus tests of any significant differences in estimated odds between the five AD dementia subgroups for the specified neuropathology outcome. While the reported *P* values have not been adjusted for multiple comparisons (5 AD models), we have bolded those that would retain significance (original α = 0.05) after applying Holm–Bonferroni adjustments for this neuropathology domain. AD, Alzheimer's disease; ADNC, Alzheimer's disease neuropathologic change; *APOE*, apolipoprotein E; CAA, cerebral amyloid angiopathy; CERAD, Consortium to Establish a Registry for Alzheimer's Disease; CI, confidence interval; df, degrees of freedom; OR, odds ratio

**FIGURE 3:**
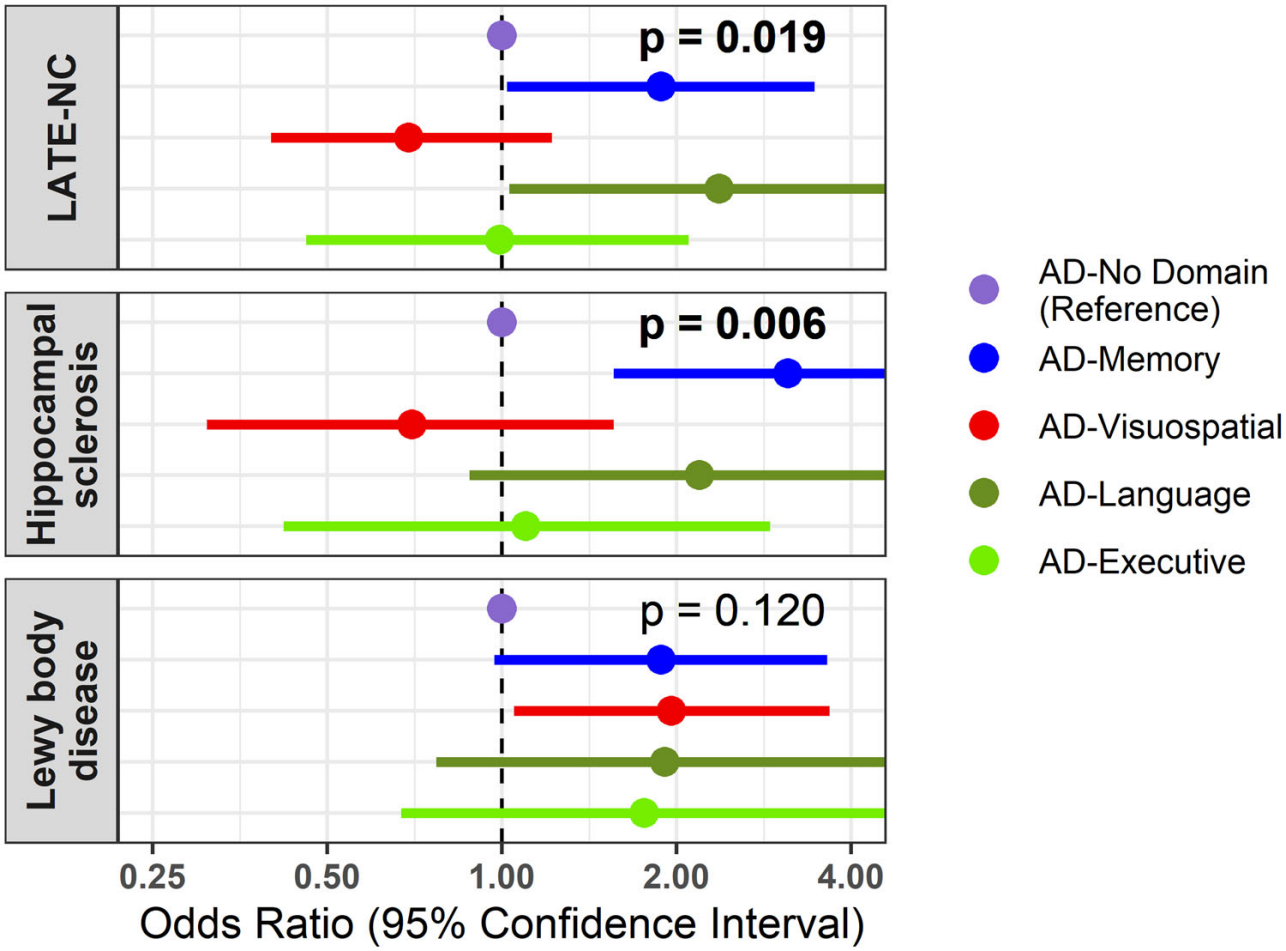
Comparisons of non‐ADNC, non‐vascular neuropathology levels across the AD dementia cognitive subgroups. OR and 95% CI are estimated from the same models used to produce Table 4 results, but results here are focused on the comparisons between AD dementia subgroups. OR axis is shown using a log scale. The reference group for all estimates shown in this figure is the AD–no domain dementia subgroup. *P* values correspond to 4‐degrees of freedom omnibus tests of any significant differences in estimated odds between the 5 AD dementia subgroups for the specified neuropathology outcome. While the reported *P* values have not been adjusted for multiple comparisons (3 non‐AD/non‐vascular models), we have bolded those that would retain significance (original α = 0.05) after applying Holm–Bonferroni adjustments for this neuropathology domain. AD, Alzheimer's disease; CI, confidence interval; LATE‐NC, limbic‐predominant age‐related transactive response DNA binding protein 43 kDa encephalopathy neuropathologic change; OR, odds ratio

**FIGURE 4:**
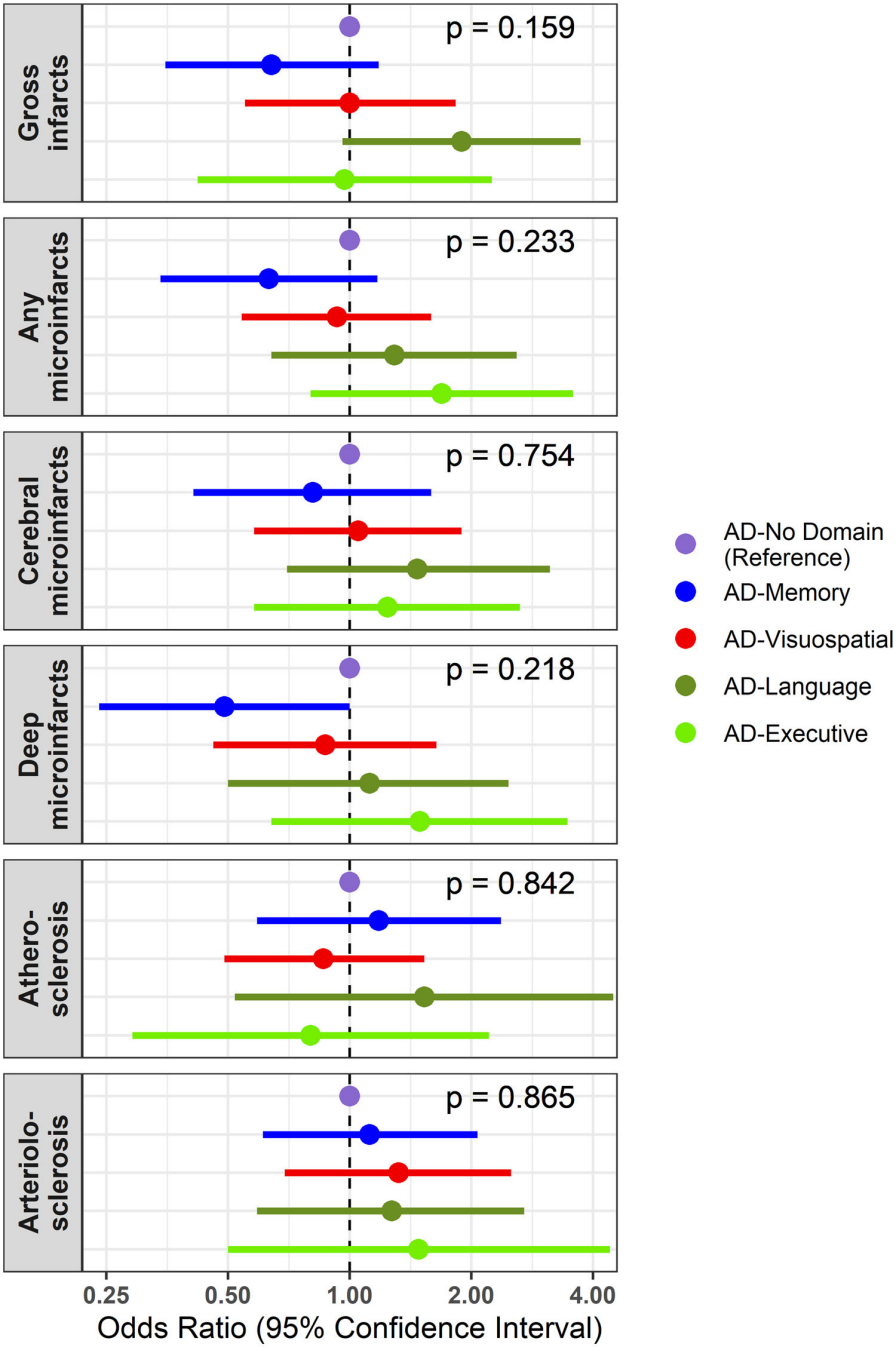
Comparisons of vascular neuropathology levels across the AD dementia cognitive subgroups. OR and 95% CI are estimated from the same models used to produce Table 4 results, but results here are focused on the comparisons between AD dementia subgroups. Odds ratio axis is shown using a log scale. The reference group for all estimates shown in this figure is the AD–no domain dementia subgroup. *P* values correspond to 4‐degrees of freedom omnibus tests of any significant differences in estimated odds between the 5 AD dementia subgroups for the specified neuropathology outcome. While the reported *P* values have not been adjusted for multiple comparisons (5 AD models), we have bolded those that would retain significance (original α = 0.05) after applying Holm–Bonferroni adjustments for this neuropathology domain. AD, Alzheimer's disease; CI, confidence interval; OR, odds ratio

For all ADNC measures (Figure 2), the AD–visuospatial subgroup consistently had the lowest estimated odds. The AD–memory subgroup had the largest estimated odds for the Thal and Braak components of the ADNC score but not for the CERAD component or for CAA. The estimates for AD–memory (relative to AD–no domain) were OR 1.64, 95% CI 0.88 to 3.05 for Thal phase and OR 2.25, 95% CI 1.22 to 4.15 for Braak stage. Unadjusted for multiple comparisons, *P* values from 4 df omnibus tests of an association between AD dementia subgroup and levels of each ADNC measure were: Thal phase (*P* = 0.087), Braak stage (*P* = 0.019), and CAA (*P* = 0.126). While the test for the Braak outcome yielded the only *P* value < 0.05, we note that its *P* value of 0.019 was slightly larger than the 0.010 threshold dictated by a Holm–Bonferroni adjustment for multiple testing across five ADNC outcome measures. Results from models without *APOE* adjustment (Figure S1) yielded similar patterns of results albeit with slightly stronger estimates of association.

For non‐ADNC, non‐vascular pathologies (Figure 3), we found evidence of an association between AD dementia subgroup and the odds of more severe pathology for LATE‐NC (*P* = 0.019) and HS (*P* = 0.006), both of which retained significance after Holm–Bonferroni adjustment for multiple testing of three outcome measures within this neuropathology domain. In both instances, the AD–visuospatial subgroup was estimated to have the lowest odds. In contrast, the highest estimated odds were for the AD–memory (LATE‐NC OR 1.88, 95% CI 1.02–3.46 and HS OR 3.11, 95% CI 1.56–6.20) and AD–language (LATE‐NC OR 2.37, 95% CI 1.03–5.46 and HS OR 2.19, 95% CI 0.88–5.50) subgroups. No significant differences were identified across AD dementia subgroups for LBD pathology, though OR point estimates comparing each single domain AD dementia subgroup to the AD–no domain subgroup were similarly elevated near 2. Models unadjusted for *APOE* (Figure S2) provided similar findings.

For vascular pathologies (Figure 4), we found no significant associations with AD subgroup (all *P* > 0.05). The AD–memory subgroup had the lowest estimated odds for the infarct outcomes, but no consistent pattern was observed across the vascular outcome measures. The same held true in models without *APOE* adjustment (Figure S3).

## 4 DISCUSSION

AD dementia is clinically heterogeneous, yet the neuropathologic correlates of this heterogeneity remain incompletely understood. In a community‐based brain donation cohort from the ACT study, cognitively defined AD subgroups had higher levels of both ADNC and non‐vascular neuropathology (LATE‐NC, HS, and LBD) than people without dementia. Across AD cognitive subgroups, we found evidence of differences in LATE‐NC and HS and consistent patterns for select ADNC measures. Although substantial overlap in pathology was observed, these findings suggest that cognitively defined subgroups may offer a useful framework for examining clinicopathologic heterogeneity, particularly for pathologies such as TDP‐43 proteinopathy for which validated in vivo biomarkers remain limited.

### 4.1 AD cognitive subgroups differ meaningfully in neuropathologic severity

Cognitive heterogeneity in AD dementia aligned with variation in Braak stage and Thal phase and was associated with LATE‐NC and HS, whereas evidence for vascular and other pathologies was lacking. Although all individuals in AD cognitive subgroups met clinical criteria for AD dementia, their neuropathologic profiles varied substantially, supporting the view that mixed pathology is common in late‐life dementia.^16^, ^18^, ^42^ These findings extend prior work showing cognitive subgroup differences in atrophy patterns, glucose hypometabolism, genetic associations, and clinical features^8^, ^9^ and support cognitive subgrouping as a framework for characterizing neuropathologic heterogeneity.

### 4.2 The AD–visuospatial subgroup exhibits relatively low ADNC burden

Odds of higher pathology levels for each ADNC outcome were consistently estimated to be lowest for AD–visuospatial. Differences with other subgroups were partially attenuated after adjustment for *APOE* ε4 carrier status, and no associations reached statistical significance. Compared to no dementia, AD–visuospatial tended to show elevated odds of ADNC (particularly for Braak stage and overall ADNC score), but associations were generally weaker than for several other AD subgroups. Thus, a visuospatial‐predominant clinical presentation can occur across a range of global ADNC burden and is not limited to individuals with the highest levels of canonical ADNC. Consensus AD staging metrics such as Thal phase and Braak stage provide global, ordinal measures rather than regionally resolved assessments and may not fully capture heterogeneity in the spatial distribution of tau and amyloid burden.^1^, ^15^ Prior neuropathologic studies demonstrate substantial heterogeneity in regional tau distribution, including hippocampal‐sparing and limbic‐predominant variants.^50^ Consistent with this literature, neuroimaging in these cognitive subgroups showed relatively greater posterior cortical atrophy in the AD–visuospatial group.^9^ Although our measures do not directly assess regional tau burden, these findings are compatible with prior evidence that regional variation in pathology contributes to heterogeneity in clinical presentation. AD–visuospatial also had the lowest estimated odds of LATE‐NC and HS, suggesting that limbic‐predominant co‐pathologies may be less prominent contributors to this phenotype.

The relationship between visuospatial impairment and Braak stage is complex. While Braak stage VI reflects involvement of primary visual cortex, visuospatial dysfunction can arise from degeneration of posterior association cortices, including parietal and occipital networks.^1^, ^51^, ^52^, ^53^, ^54^ Accordingly, global staging frameworks, like Braak, do not distinguish regional patterns that may be clinically relevant to visuospatial function. This interpretation aligns with tau positron emission tomography studies showing that visuospatial performance and posterior cognitive dysfunction are more closely associated with tau burden in parietal and occipital association cortex than overall Braak stage.^55^ Thus, lower representation of the highest Braak stages in AD–visuospatial does not preclude posterior‐predominant network involvement, which cannot be directly evaluated with staging measures used here.

### 4.3 AD–memory subgroup shows higher burden of canonical ADNC and limbic co‐pathology

Although *APOE* ε4–adjusted associations with ADNC outcomes did not reach statistical significance, AD–memory had the highest estimated odds for Thal phase, Braak stage, and overall ADNC score, positioning AD–memory closest to a classical limbic‐predominant ADNC profile. In parallel, AD–memory also showed higher estimated burdens of LATE‐NC and HS, consistent with predominant involvement of medial temporal structures supporting episodic memory.^18^, ^19^, ^43^, ^56^ These findings align with recent LATE clinical criteria identifying amnestic AD patients with disproportionate hippocampal atrophy or memory‐specific deficits as having elevated likelihood of LATE.^28^ However, LATE‐NC occurred across multiple AD cognitive subgroups, underscoring that it is not specific to AD–memory.

### 4.4 AD–language subgroup shows higher estimated odds of LATE and hippocampal sclerosis

The AD–language subgroup also tended to demonstrate higher estimated odds of more severe LATE‐NC and HS. Although LATE‐NC is commonly associated with memory impairment, involvement beyond medial temporal structures may affect temporal association cortices critical for language processing, including semantic and lexical functions.^27^, ^57^ These findings suggest that LATE‐NC and related limbic pathology may contribute to a language‐predominant presentation in some individuals.^19^, ^58^, ^59^, ^60^, ^61^

Although between‐subgroup differences in LATE‐NC and HS were most pronounced for AD–memory and AD–language, nearly every AD subgroup showed elevated estimated odds relative to no dementia. LATE‐NC ORs ranged from 1.19 (95% CI 0.65–2.18) in AD–visuospatial to 4.07 (95% CI 1.70–9.76) in AD–language (Table 4). HS estimates ranged from 1.68 (95% CI 0.67–4.24) in AD–visuospatial to 7.49 (95% CI 3.33–16.85) in AD–memory. In AD–visuospatial and AD–executive subgroups most ORs remained elevated, with wide CIs, consistent with limited power. These patterns suggest that LATE‐NC and HS contribute broadly to AD dementia while varying by cognitive phenotype. Similarly, arteriolosclerosis estimates were elevated across all AD subgroups relative to no dementia (ORs ranging from 1.33–1.97; Table 4), despite no significant between‐subgroup differences (Figure 4), suggesting that some co‐pathologies may be general contributors rather than phenotype‐specific contributors.

### 4.5 Lewy body co‐pathology and cerebrovascular findings across AD cognitive subgroups

Beyond ADNC and LATE‐NC, we observed associations with Lewy body co‐pathology and gross infarcts. Relative to no dementia, AD–memory, AD–visuospatial, and the AD–language subgroups had higher estimated odds of more anatomically widespread Lewy body pathology (*APOE*‐adjusted ORs [95% CIs]: 2.58 [1.24–5.36], 2.69 [1.33–5.45], and 2.61 [1.01–6.75], respectively; omnibus *P* = 0.017; Table 4). The distribution of LBD subtypes provides further anatomic context for these associations (Table 3); neocortical LBD was most prevalent in AD–visuospatial (20%) and AD–language (23%),^23^ consistent with involvement of cortical networks supporting visuospatial and language function, whereas AD–memory was enriched for limbic and amygdala‐predominant LBD, consistent with medial temporal involvement.

AD–language, AD–visuospatial, and AD–no domain had the highest estimated odds of gross infarcts relative to no dementia (*APOE*‐adjusted ORs: 4.23 [1.97–9.09], 2.24 [1.16–4.30], and 2.24 [1.35–3.70], respectively; omnibus *P* = 0.004; Table 4), while the AD–memory subgroup had the lowest estimated odds (OR 1.43 [0.73–2.80]). In descriptive summaries of infarct locations, cortical infarcts were more common in AD–language and rare in AD–visuospatial, while white matter infarcts were less common in AD–memory (Table S3 in supporting information). Larger studies with sufficient power to formally test infarct location associations will be needed to determine whether these descriptive patterns reflect meaningful clinicopathologic relationships.

Although omnibus tests for microinfarcts did not reach statistical significance (any microinfarcts *P* = 0.081; deep microinfarcts *P* = 0.054), the AD–executive subgroup showed the highest estimated odds for both outcomes (OR 2.78 [1.24–6.21] and 2.96 [1.17–7.50], respectively). This pattern is biologically plausible given the established role of subcortical microvascular disease in executive dysfunction,^62^ and warrants replication.

### 4.6 Strengths and limitations

A major strength of the study is integration of deep, longitudinal cognitive characterization with high‐quality neuropathologic evaluation in a large, community‐based brain donation cohort, supporting population‐level inference while minimizing specialty‐clinic biases. Simultaneous evaluation of ADNC, LATE‐NC, HS, vascular injury, and Lewy body pathology enabled assessment of mixed pathologies across cognitive phenotypes using standardized consensus criteria.

Limitations include variability in the interval between clinical diagnosis and death, potentially creating mismatch between subgroup assignment and neuropathology at death, and limited sample sizes for some subgroups (Table 1). Global, ordinal staging systems may also miss regional or network‐level pathology relevant to cognitive subgroups. Finally, while differential patterns of co‐pathology were observed, the data and study design precludes causal or temporal inference, and most subgroup‐specific patterns did not meet corrected significance thresholds; findings should therefore be interpreted as exploratory.

### 4.7 Future directions

Future studies incorporating quantitative, regionally resolved neuropathologic measures will clarify subgroup‐specific patterns of pathology and network vulnerability. Expanded regional sampling beyond traditional staging frameworks may further refine clinicopathologic associations in cognitively defined AD subgroups.

Motor and movement function were not incorporated into our cognitive subgrouping framework. Corticobasal syndrome (CBS) can arise with high‐level ADNC, and ADNC‐confirmed CBS cases disproportionately exhibit an idiotypic‐susceptible NFT subtype characterized by elevated tangle burden in primary motor and somatosensory cortices relative to hippocampus and association cortex.^63^ Extending subgrouping to motor and praxis assessments may identify a corresponding clinical phenotype.

The overarching objective of this study was to identify neuropathologic features that differentiate cognitively defined subgroups within clinically diagnosed AD dementia. We found evidence of differences in LATE‐NC and HS across select AD cognitive subgroups and patterns in some ADNC outcomes. Individuals in the AD–memory subgroup tended to exhibit higher levels of canonical ADNC and LATE‐NC; the AD–visuospatial subgroup tended to show lower estimated ADNC burdens and LATE‐NC; and the AD–language subgroup showed higher estimated odds of more advanced LATE pathology and HS. Collectively, these findings suggest that cognitive heterogeneity in AD dementia is associated with differences in the relative burden of ADNC and select co‐pathologies, although substantial overlap exists across subgroups. These results may inform future efforts to better characterize heterogeneity in AD dementia.

## FUNDING INFORMATION

Funding from the National Institute on Aging includes K08AG065426 (C.S.L.); P30AG066509 (C.S.L., C.D.K.); U19AG066567 (C.S.L., R.L.W., C.D.K., S.M., S.E.C., M.L.L., C.N., K.E., L.K.M., and P.K.C.); U01AG006781 (prior funding that additionally supported data collection); additional funding includes the Nancy and Buster Alvord endowment (C.D.K.). All statements in this manuscript, including its findings and conclusions, are solely those of the authors and do not necessarily represent the views of the National Institute on Aging or the National Institutes of Health.

## CONFLICT OF INTEREST STATEMENT

The authors declare no conflicts of interest. Author disclosures are available in the Supporting Information.

## CONSENT STATEMENT

All human subjects provided informed consent.

## Supporting information



Supporting Information

Supporting Information

Supporting Information

Supporting Information

Supporting Information

Supporting Information

Supporting Information
