# Supplementary material for: Neuropathologic features of cognitively defined Alzheimer's disease subgroups in the Adult Changes in Thought study
Source: Alzheimers Dement. 2026 Oct 25;22(10):e71938. doi: 10.1002/alz.71938 (PMC13696749; doi:10.1002/alz.71938)
Supplement: Supplementary file 3 [file ALZ-22-e71938-s002.docx]

**Supplemental Fig. S3 Comparisons of vascular neuropathology levels across the AD dementia cognitive subgroups, without adjustment for APOE.**

Odds ratios and 95% confidence intervals are estimated from the same models used to produce Supplemental Table S2 results, but results here are focused on the comparisons between AD dementia subgroups. Odds ratio axis is shown using a log scale. The reference group for all estimates shown in this figure is the AD-No Domain dementia subgroup. P-values correspond to 4-degrees of freedom omnibus tests of any significant differences in estimated odds between the 5 AD dementia subgroups for the specified neuropathology outcome. While the reported p-values have not been adjusted for multiple comparisons (5 AD models), we have bolded those that would retain significance (original α=0.05) after applying Holm-Bonferroni adjustments for this neuropathology domain.

Abbreviations: AD, Alzheimer’s disease; APOE, apolipoprotein E; OR, odds ratio; CI, confidence interval; df, degrees of freedom; ADNC, Alzheimer’s disease neuropathologic change; CERAD, Consortium to Establish a Registry for Alzheimer's Disease; CAA, cerebral amyloid angiopathy
