# Supplementary material for: Neuropathologic features of cognitively defined Alzheimer's disease subgroups in the Adult Changes in Thought study
Source: Alzheimers Dement. 2026 Oct 25;22(10):e71938. doi: 10.1002/alz.71938 (PMC13696749; doi:10.1002/alz.71938)
Supplement: Supplementary file 4 [file ALZ-22-e71938-s003.docx]

**Supplemental Table S2.** Comparisons of neuropathology levels: 1.) between participants with non-AD dementia and participants without dementia; and 2.) between AD dementia cognitive subgroups and participants without dementia. **Estimates not adjusted for *APOE***.

| **Neuropathology** | **Non-AD dementia vs.**  **No dementia**  **OR (95% CI)^a^** | **Each AD dementia cognitive subgroup vs. No dementia** | | | | | |
| --- | --- | --- | --- | --- | --- | --- | --- |
|  |  | **AD-**  **No Domain**  **OR (95% CI)** | **AD-**  **Memory**  **OR (95% CI)** | **AD-**  **Visuospatial**  **OR (95% CI)** | **AD-**  **Language**  **OR (95% CI)** | **AD-**  **Executive**  **OR (95% CI)** | **Omnibus p-value^b^** |
| **ADNC measures** |  |  |  |  |  |  |  |
| ADNC score | **2.56 (1.48, 4.43)** | **3.58 (2.21, 5.79)** | **8.43 (4.07, 17.46)** | **2.57 (1.32, 5.02)** | **4.53 (2.05, 10.00)** | **4.45 (1.69, 11.69)** | <0.001* |
| Thal phase | **1.84 (1.03, 3.28)** | **2.23 (1.35, 3.68)** | **4.14 (2.00, 8.59)** | 1.10 (0.61, 1.98) | **2.38 (1.13, 5.03)** | 2.04 (0.82, 5.05) | 0.001* |
| Braak stage | **2.80 (1.77, 4.45)** | **4.20 (2.69, 6.55)** | **9.54 (4.87, 18.68)** | **2.62 (1.40, 4.91)** | **5.87 (2.69, 12.78)** | **5.35 (2.25, 12.73)** | <0.001* |
| CERAD score | **2.10 (1.20, 3.68)** | **2.81 (1.76, 4.50)** | **3.84 (2.02, 7.28)** | 1.61 (0.87, 3.00) | **3.76 (1.77, 7.97)** | **2.41 (1.06, 5.49)** | <0.001* |
| CAA | 1.02 (0.58, 1.78) | 1.03 (0.64, 1.65) | 1.73 (0.96, 3.11) | 0.63 (0.34, 1.17) | 1.07 (0.47, 2.46) | 1.72 (0.73, 4.06) | 0.085 |
| **Non-ADNC, non-vascular pathology** |  |  |  |  |  |  |  |
| LATE-NC | 0.92 (0.53, 1.60) | **1.73 (1.07, 2.77)** | **3.46 (1.80, 6.67)** | 1.15 (0.63, 2.09) | **3.72 (1.58, 8.72)** | 1.57 (0.70, 3.50) | 0.001* |
| Hippocampal sclerosis | 1.97 (0.79, 4.89) | **2.42 (1.19, 4.90)** | **7.83 (3.46, 17.70)** | 1.63 (0.65, 4.09) | **5.08 (1.74, 14.80)** | 2.56 (0.87, 7.54) | <0.001* |
| Lewy body disease | 1.80 (0.97, 3.33) | 1.40 (0.77, 2.52) | **2.70 (1.31, 5.57)** | **2.60 (1.28, 5.28)** | **2.55 (1.00, 6.50)** | 2.24 (0.81, 6.20) | 0.021* |
| **Vascular pathology** |  |  |  |  |  |  |  |
| Gross infarcts | **3.75 (2.20, 6.40)** | **2.25 (1.36, 3.71)** | 1.45 (0.74, 2.83) | **2.23 (1.16, 4.27)** | **4.17 (1.96, 8.87)** | 2.14 (0.87, 5.27) | 0.003* |
| Any microinfarcts | **2.48 (1.38, 4.46)** | 1.64 (1.02, 2.62) | 1.00 (0.52, 1.91) | 1.54 (0.87, 2.76) | 2.16 (1.01, 4.62) | 2.85 (1.27, 6.37) | 0.057 |
| Cerebral microinfarcts | **2.25 (1.29, 3.93)** | 1.38 (0.83, 2.29) | 1.10 (0.55, 2.20) | 1.45 (0.78, 2.73) | 2.06 (0.91, 4.64) | 1.73 (0.77, 3.89) | 0.507 |
| Deep microinfarcts | **2.83 (1.47, 5.43)** | 1.98 (1.15, 3.40) | 0.94 (0.43, 2.04) | 1.75 (0.86, 3.56) | 2.27 (0.95, 5.43) | 3.08 (1.21, 7.81) | 0.039 |
| Atherosclerosis | 1.87 (0.99, 3.52) | 1.20 (0.73, 1.98) | 1.38 (0.67, 2.84) | 1.05 (0.57, 1.95) | 1.90 (0.62, 5.81) | 1.01 (0.36, 2.82) | 0.857 |
| Arteriolosclerosis | **2.86 (1.49, 5.51)** | 1.33 (0.78, 2.27) | 1.48 (0.74, 2.98) | 1.76 (0.86, 3.61) | 1.69 (0.73, 3.91) | 1.98 (0.64, 6.13) | 0.587 |

Odds ratios and 95% confidence intervals are estimated from ordinal logistic regression models – a separate model for each neuropathology outcome – adjusted for sex, age at death, years of education, and years from visit to death, and incorporating inverse-probability weighting to account for selection into the analytic sample. The reference group for all estimates shown in this table is the No dementia group. ORs>1 correspond to higher estimated levels of the neuropathology outcome in the specified group (relative to No dementia).

^a^ For the Non-AD dementia vs. No dementia comparisons, estimates for which the 95% CI excludes 1 are in **bold**.

^b^ P-values correspond to 5-degrees of freedom omnibus tests of any significant differences in estimated odds between the 5 AD dementia subgroups and the No dementia group for the specified neuropathology outcome. While the reported p-values have not been adjusted for multiple comparisons (5 AD models, 3 non-AD / non-vascular models, and 6 vascular models), we have denoted with an asterisk (*) those that retain significance (original α=0.05) after applying Holm-Bonferroni adjustments separately per neuropathology domain; only in those instances do we additionally **bold** any corresponding AD dementia subgroup pairwise estimates for which the original 95% CI excludes 1.

Abbreviations: AD, Alzheimer’s disease; APOE, apolipoprotein E; OR, odds ratio; CI, confidence interval; df, degrees of freedom; ADNC, Alzheimer’s disease neuropathologic change; CERAD, Consortium to Establish a Registry for Alzheimer's Disease; CAA, cerebral amyloid angiopathy; LATE-NC, limbic-predominant age-related TDP-43 encephalopathy neuropathologic change
