# Supplementary material for: Neuropathologic features of cognitively defined Alzheimer's disease subgroups in the Adult Changes in Thought study
Source: Alzheimers Dement. 2026 Oct 25;22(10):e71938. doi: 10.1002/alz.71938 (PMC13696749; doi:10.1002/alz.71938)
Supplement: Supplementary file 5 [file ALZ-22-e71938-s005.docx]

**Supplemental Table S1.** Characteristics of all ACT participants ever enrolled and potentially eligible for analysis^a^, the subset of participants who consented to brain donation, and the final analytic cohort with post-mortem neuropathology data included in this manuscript.

| **Characteristics** | **All ACT participants**^a^  **(n=5729)** | **Consented to brain donation**  **(n=2045)** | **Final analytic cohort**  **(n=885)** |
| --- | --- | --- | --- |
|  |  |  |  |
| Women | 3324 (58%) | 1204 (59%) | 512 (58%) |
| Non-Hispanic White^b^ | 5091 (89%) | 1871 (91%) | 828 (94%) |
| Years of biennial visit follow-up | 8.9 (6.2) | 10.2 (6.0) | 10.1 (6.0) |
| Age at visit | 83.0 (7.2) | 84.4 (6.9) | 86.3 (6.5) |
| Years of education | 15.0 (3.2) | 15.6 (3.1) | 14.9 (3.0) |
| Cognitive status |  |  |  |
| No dementia | 4250 (74%) | 1379 (67%) | 438 (49%) |
| AD dementia^c^ | 1214 (21%) | 552 (27%) | 370 (42%) |
| Non-AD dementia^d^ | 265 (5%) | 114 (6%) | 77 (9%) |
| At least one *APOE* ε4 allele^e^ | 1237 (26%) | 483 (26%) | 256 (29%) |
| Coronary artery disease | 1500 (26%) | 526 (26%) | 272 (31%) |
| Cerebrovascular disease | 1159 (20%) | 473 (23%) | 258 (29%) |
| # of ADLs with difficulty^f^ | 1.0 (1.5) | 1.1 (1.6) | 1.5 (1.8) |
| Unable to perform timed walking task^g^ | 158 (4%) | 77 (5%) | 45 (7%) |

Data provided in this table are from the biennial study visit at which participants were diagnosed with dementia (of any type) or from the latest study visit for those who were not diagnosed with dementia. Values shown in this table are either n (%) or mean (standard deviation) depending on the characteristic.

^a^ After excluding participants in the AD-Multiple Domain subgroup (N=34) per Figure 1.

^b^ Due to small proportions of participants in most race/ethnicity categories, we only report the proportion of participants categorized as non-Hispanic White, which was the most common category.

^c^ Participants who met NINCDS-ADRDA criteria for AD.

^d^ Participants diagnosed with dementia per DSM-IV criteria but who did not meet NINCDS-ADRDA criteria for AD.

^e^ APOE information was unavailable for 1010 (18%) of all ACT participants and 216 (11%) of participants who consented to brain donation.

^f^ ADL information was unavailable for 315 (5%) of all ACT participants, 124 (6%) of participants who consented to brain donation, and 74 (8%) of participants in the final analytic cohort.

^g^ The 10-foot timed walking task was not assessed for 1495 (26%) of all ACT participants, 520 (25%) of participants who consented to brain donation, and 225 (25%) of participants in the final analytic cohort; therefore, % unable calculated among those assessed.

Abbreviations: AD, Alzheimer’s disease; APOE, apolipoprotein E; NINCDS-ADRDA, National Institute of Neurological and Communicative Disorders and Stroke and Alzheimer's Disease and Related Disorders Association; DSM-IV, Diagnostic and Statistical Manual of Mental Disorders, Fourth Edition
