# Supplementary material for: Neuropathologic features of cognitively defined Alzheimer's disease subgroups in the Adult Changes in Thought study
Source: Alzheimers Dement. 2026 Oct 25;22(10):e71938. doi: 10.1002/alz.71938 (PMC13696749; doi:10.1002/alz.71938)
Supplement: Supplementary file 7 [file ALZ-22-e71938-s007.docx]

**Supplemental Table S3.** Distribution of gross infarcts by location, overall and stratified by dementia and AD dementia cognitive subgroup classification.

| **Number of gross infarcts**^a^ | **Total**  **(n=885)** | **No dementia**  **(n=438)** | **Non-AD dementia**  **(n=77)** | **AD dementia** | | | | |
| --- | --- | --- | --- | --- | --- | --- | --- | --- |
|  |  |  |  | **AD-**  **No Domain**  **(n=201)** | **AD-**  **Memory**  **(n=56)** | **AD-**  **Visuospatial**  **(n=55)** | **AD-**  **Language**  **(n=31)** | **AD-**  **Executive**  **(n=27)** |
| Cortical |  |  |  |  |  |  |  |  |
| 0 | 767 (87%) | 397 (91%) | 62 (82%) | 167 (84%) | 47 (84%) | 52 (95%) | 18 (58%) | 24 (89%) |
| 1 | 78 (9%) | 27 (6%) | 8 (11%) | 25 (12%) | 7 (12%) | 1 (2%) | 8 (26%) | 2 (7%) |
| 2 | 21 (2%) | 4 (1%) | 5 (7%) | 5 (2%) | 2 (4%) | 1 (2%) | 3 (10%) | 1 (4%) |
| 3+ | 17 (2%) | 10 (2%) | 1 (1%) | 3 (2%) | 0 (0%) | 1 (2%) | 2 (6%) | 0 (0%) |
| White matter |  |  |  |  |  |  |  |  |
| 0 | 784 (89%) | 408 (93%) | 63 (83%) | 167 (84%) | 54 (96%) | 48 (87%) | 22 (71%) | 22 (81%) |
| 1 | 72 (8%) | 23 (5%) | 10 (13%) | 22 (11%) | 1 (2%) | 6 (11%) | 6 (19%) | 4 (15%) |
| 2 | 15 (2%) | 4 (1%) | 1 (1%) | 8 (4%) | 0 (0%) | 1 (2%) | 1 (3%) | 0 (0%) |
| 3+ | 12 (1%) | 3 (1%) | 2 (3%) | 3 (2%) | 1 (2%) | 0 (0%) | 2 (6%) | 1 (4%) |
| Deep gray matter / internal capsule |  |  |  |  |  |  |  |  |
| 0 | 717 (81%) | 383 (87%) | 52 (68%) | 158 (79%) | 46 (82%) | 39 (71%) | 19 (61%) | 20 (74%) |
| 1 | 104 (12%) | 34 (8%) | 14 (18%) | 25 (12%) | 7 (12%) | 8 (15%) | 11 (35%) | 5 (19%) |
| 2 | 31 (4%) | 9 (2%) | 5 (7%) | 11 (6%) | 1 (2%) | 4 (7%) | 0 (0%) | 1 (4%) |
| 3+ | 31 (4%) | 12 (3%) | 5 (7%) | 6 (3%) | 2 (4%) | 4 (7%) | 1 (3%) | 1 (4%) |
| Brainstem / cerebellum |  |  |  |  |  |  |  |  |
| 0 | 796 (90%) | 401 (92%) | 66 (87%) | 179 (90%) | 50 (89%) | 48 (87%) | 27 (87%) | 25 (93%) |
| 1 | 69 (8%) | 28 (6%) | 8 (11%) | 14 (7%) | 6 (11%) | 7 (13%) | 4 (13%) | 2 (7%) |
| 2 | 13 (1%) | 8 (2%) | 1 (1%) | 4 (2%) | 0 (0%) | 0 (0%) | 0 (0%) | 0 (0%) |
| 3+ | 5 (1%) | 1 (0%) | 1 (1%) | 3 (2%) | 0 (0%) | 0 (0%) | 0 (0%) | 0 (0%) |

^a^ Gross infarcts were unavailable for 2 (0.2%) of participants.

Abbreviations: AD, Alzheimer’s disease
